# Experiences, views and perceptions of recovery following musculoskeletal trauma of patients and physiotherapists: a qualitative study

**DOI:** 10.1371/journal.pone.0323575

**Published:** 2025-05-28

**Authors:** Nicola Middlebrook, Nicola R. Heneghan, Maria Moffatt, Lucy Silvester, Deborah Falla, Alison B. Rushton, Andrew A. Soundy

**Affiliations:** 1 Department of Health Professions, Faculty of Health and Education, Manchester Metropolitan University, Manchester, United Kingdom; 2 School of Sport, Exercise and Rehabilitation Sciences, College of Life and Environmental Sciences, University of Birmingham, Birmingham, United Kingdom; 3 School of Allied Health Professions and Nursing, Institution of Population Health, University of Liverpool, Liverpool, United Kingdom; 4 Institute for Applied & Translational Technologies in Surgery, University Hospitals Coventry and Warwickshire NHS Trust, Clifford Bridge Road, Coventry, United Kingdom; 5 Western University, School of Physical Therapy, London, Ontario, Canada; 6 Centre of Precision Rehabilitation for Spinal Pain, School of Sport, Exercise and Rehabilitation Sciences, College of Life and Environmental Sciences, University of Birmingham, Birmingham, United Kingdom; University of Minnesota, UNITED STATES OF AMERICA

## Abstract

The aim of this qualitative phenomenology study using two methods (semi-structured interviews, focus groups), was to explore patients’ and physiotherapists’ views and perceptions of recovery, and what constitutes successful recovery following musculoskeletal trauma within the early stages of recovery. Participants were recruited from one major trauma centre in the United Kingdom and data collected via Microsoft Teams, or via a telephone call. Inclusion criteria for patient interviews: purposive sample of adults (≥18 years) who sustained a traumatic musculoskeletal injury, admitted as an inpatient within 4 weeks of injury, mental capacity, and able to communicate in English. Purposive sampling included age, gender, and injury characteristics. Focus group inclusion criteria: physiotherapists with experience managing patients with musculoskeletal trauma. Interviews and focus groups were informed by an evidenced based topic guide, audio recorded and transcribed verbatim. Trustworthiness of the data was strengthened using multiple strategies, e.g., member checking. Interpretative Phenomenological Analysis was used for the patient interviews and the Kreuger Framework for the focus groups. Participants included 17 patient interviews and 10 physiotherapists in two focus groups. Three themes emerged from patient interviews: understanding and impact of the accident and injuries, the early stages of recovery and physiotherapy, and healthcare and setting influences. Eight themes emerged from the focus groups: process of recovery, what is being fully recovered, it’s more than just communicating with the patient, psychological impact of trauma affecting recovery, system influences/resources for recovery, influencers to recovery, barriers to using patient reported outcome measures to evaluate recovery, and what actually is useful to measure in trauma? Recovery following musculoskeletal trauma is complex, individual and focused on returning to ‘normal’. Similarities across patient and physiotherapist views of recovery exist. Differences between participant groups were evident, centred on communication and what is important to the patient in their recovery.

## Introduction

Injuries following a traumatic event such as a fall or road traffic accident are common globally [1]. Annually, it is estimated up to 500,000 people are involved in a traumatic accident in the UK [2–4], and with advances in healthcare more people are now surviving complex injuries [5]. Patients often require complex rehabilitation beyond 12 months, with 30–40% of survivors not returning to their pre-injury level of work and daily activities within 12–24 months following injury [6–8]. High pain intensity at injury, psychological factors such as post-traumatic stress disorder (PTSD), reduced self-efficacy, and education levels have been suggested as prognostic factors associated with recovery [6,9–12]. Most of these studies [6,9–12] use pain as a measure of recovery, with ‘successful recovery’ not well defined within the literature. Various other outcome measures have also been used to define and measure recovery, including generic health related questionnaires such as SF-36, EuroQol, the Chronic Pain Grade Scale, return to work status and injury severity.

Current National Institute for Clinical Excellence (NICE) guidelines recommend a person-centred, individualised and holistic approach to optimise rehabilitation and recovery [4]. Physiotherapists are integral to rehabilitation aiming to reduce pain and restore function, and are key in signposting to other services and professionals where required [4].

Little is known about the patient perspective on recovery, what is important during their recovery, and what successful recovery means to them. This raises the question of whether we are using the most appropriate outcome measures to monitor recovery progress and highlights the need to explore the patients’ views on recovery. A recent qualitative systematic review aimed to synthesise current studies on patient perspectives of recovery [13]. This review included studies ranging from an early recovery to long-term recovery of up to 17 years. However, the studies which focused on early recovery (defined as less than 6 months) varied in methodological quality or were focused on very specific populations, e.g., tibial fractures or hand injuries. Furthermore, only one explored recovery within 35 days of injury, and this included just participants with lower limb fractures [14]. Systematic review findings highlighted the complexity of recovery with different themes emerging such as adapting and learning to manage their injuries [13]. However, no study explores the patient views and experiences at the very early stages following injury for all musculoskeletal trauma warranting further investigation. Evidence suggests that whilst distress following injury is a normal and expected response [15], prolonged distress in the weeks following the injury such as anxiety, catastrophic thinking, can predict poorer outcome [6,16–18]. It is vital to capture patient views and lived experiences at the early stage of recovery to understand how healthcare can support patients during this crucial time following injury when interactions with healthcare are at their highest. With a better understanding of the early recovery following the injury, there is potential to target interactions and rehabilitation interventions that are more patient centered, and shape recovery at an early stage, enabling a better return to function/return to work long term.

Physiotherapists are integral to rehabilitation following injury. However previous research focused on shoulder problems has found that physiotherapist views on recovery may not fully align with patient views [19]. To the authors knowledge no study has explored the views of physiotherapists on recovery and successful recovery in musculoskeletal trauma. It is imperative to not only gain the views of patients around recovery but to understand the views of physiotherapists on recovery, how they measure recovery, and whether this aligns with patient views.

### Aim

To explore patients’ and physiotherapists’ views and perceptions of recovery, and what constitutes successful recovery in the early stages following musculoskeletal trauma.

### Objectives

1. To understand and explore the initial stages of the patient journey following musculoskeletal trauma
2. To explore patients’ views and perceptions on the definition of successful recovery
3. To explore the physiotherapists’ views and perceptions of what they define as a successful patient recovery.
4. To explore views and perceptions of physiotherapists regarding outcome measures that are useful to assess recovery.

## Methods

### Design

A qualitative study was designed and undertaken using two complimentary methodologies according to a pre-defined and published protocol [20]. Both methodologies were situated within the same world view; Interpretative Phenomenological Analysis (IPA) with semi-structured interviews, [21], and the Krueger framework using interpretive hermeneutic phenomenology for the focus groups [22,23]. The reasons for and differences in these approaches can be seen in previous worked examples [24]. A critical reason for using different approaches was due to the inability to achieve specific outcomes of IPA from the focus group data, e.g., double hermeneutic. A world view of minimal hermeneutic realism was assumed. This view identifies that an external reality exists but the exact meaning is provided or constructed by an individual, in other words it is the encountering of facts which provides meaning to them [25]. This approach was taken and deemed important to undertake focus groups rather than individual interviews for the physiotherapists to gather multiple perspectives, generate discussion and monitor interactions rather than gaining individual perspectives in an interview [26]. Focus groups allow discussion and participants to share experiences and understanding which adds further meaning to the topic than semi-structured interviews alone, and has been used previously with IPA [27]. This study is reported according to Consolidated Criteria for Reporting Qualitative Studies (COREQ) and Standards for Reporting Qualitative Research [28,29].

### Setting

Patients and physiotherapists were recruited from one Major Trauma NHS Trust Site within the United Kingdom. The Major Trauma Centre is the centre for Central England Trauma Network and serves a population of more than 1.9 million which has a mixture of both urban and rural areas. The area has a combination of some of the most affluent areas within the United Kingdom but also areas of deprivation with a broad range of ethnicities. Patients are accepted by road and helicopter and have access to therapies including physiotherapy, occupational therapy, speech and language and psychology assistants. Upon discharge they have access to the Major Trauma Signposting Network and can be referred to their local hospital, community teams or outpatient physiotherapy. This service provision will vary depending on the area and resources within the local teams.

Due to restrictions imposed by COVID-19, the patient interviews were conducted online via Microsoft Teams or a telephone call. The physiotherapist focus groups were conducted online via Microsoft Teams.

### Ethical approval

This study was reviewed by London - Fulham Research Ethics Committee within the UK Health Departments’ Ethics Service and has gained approval from the Health Research Authority (HRA) (IRAS 287781/REC 20/PR/0712). The Research and Development team at the site approved and supported the study throughout. A participant information sheet was provided and written informed consent was obtained for all participants. Recruitment took place from Monday 6^th^ September 2021 to Friday 16^th^ December 2022.

### Participants

#### Patient interviews.

Small sample sizes are traditionally used with IPA to achieve a rich detailed interpretive accounts with a homogenous population [30]. Past studies have however successfully employed IPA with up to 48 participants [31]. Acknowledging that the musculoskeletal trauma population can be heterogenous in terms of injury type, severity and location of injury etc, a higher sample size of 20 was sought to account for heterogeneity and ensure the breadth of the population was well represented in the study. A purposive sample was used to ensure a range of patient characteristics including age, gender, and injury characteristics including injury location, multiple versus single injuries and fractures versus soft tissue injuries were included.

Potential participants were identified through admissions lists of the major trauma and orthopaedic wards by a team of research nurses supported by the principal investigator (LS) using the pre-defined eligibility criteria. Participants were given a Participant Information Sheet (PIS) and the research nurse team returned within 24 hours and gained informed written consent for those willing to participate in the study. Participant contact details were then passed to lead researcher (NM) to organise the first interview. Recruitment continued until the Study Management Team (AR, AS, DF NH, NM) agreed that rich insight had been reached, and further recruitment would not add to understanding.

#### Physiotherapist focus groups.

A purposive sample of physiotherapists was recruited to capture both inpatient and outpatient physiotherapists with a range of clinical experience. This allows naturalisation and generalisation to the wider physiotherapy population [32]. A target of 10–12 physiotherapists was sought with the aim of completing 2 focus groups. Potential participants were identified by team leads and invited to participate in the focus groups. If interested, they were given the PIS and were asked to contact the lead researcher (NM). Written informed consent was obtained for all participants.

### Eligibility criteria

#### Patient interviews.

**Inclusion Criteria:** Adults (≥18 years) who sustained a musculoskeletal injury from a traumatic event and were admitted to the major trauma/orthopaedic ward within 4 weeks of injury, mental capacity in order to give consent (score of more than 6 on the Abbreviated Mental Test) [33], and able to communicate in English.

**Exclusion Criteria:** Injury from a non-traumatic event or where the primary injury was a traumatic brain injury, spinal cord injury or neurological injury due to the different clinical pathway and recovery trajectory in which these conditions have.

#### Physiotherapist focus groups.

**Inclusion Criteria:** Any Health and Care Professional Council (HCPC) registered physiotherapist who was involved in the management of musculoskeletal trauma patients within the recruiting Major Trauma Centre.

**Exclusion Criteria:** No exclusion criteria

### Data collection methods

#### Patient interviews.

Data was collected using in-depth semi structured interviews within 4 weeks of injury. The interviews were based on a topic guide developed by the research team and patient co-investigator using knowledge from a previous study [6] and informed by the International Classification of Function, Disability and Health (ICF) domains [34]. The majority of interviews (n = 10) were completed by the lead researcher (NM) with MM completing a small proportion (n = 7) whilst the lead researcher was on maternity leave. NM sought training in IPA approach from experienced researchers (AS, AR) and completed pilot interviews with the study patient co-investigator and two further cognitive interviews with patients who had experienced musculoskeletal trauma (members of the Centre of Precision Rehabilitation for Spinal Pain (CPR Spine) register/PPI group) prior to data collection. MM was experienced in conducting semi-structured interviews. All interviews were audio recorded and transcribed verbatim. Field notes and a reflexive diary was utilised to enhance trustworthiness of the data. The process of member checking was employed which allowed all participants the opportunity to review the transcript and add any additional comments and further insight [35].

#### Physiotherapist focus groups.

Data was collected using focus groups and led by the lead researcher (NM) and supported by a moderator (NH) who is experienced in conducting focus groups. Prior to the focus groups, NM sought training from experienced researchers (NH/AS/AR). The focus groups were based on a topic guide developed by the research team which aligned to the semi-structured interview topic guide to ensure similar topics were covered. NH was present for the focus groups and took field notes and monitored progress. All focus groups were audio recorded and transcribed verbatim. All participants were invited to review the transcript following the focus group and to add any additional comments/further insights.

### Data analysis

#### Patient interviews.

Analysis consisted of four stages using IPA [36]

1. First read of transcripts by NM and MM independently.
2. Preliminary themes identified by NM and MM independently and coded in accordance with IPA. Discussion of preliminary themes with AS who acted as a critical friend during initial coding and presented to co-investigators
3. NM grouped emerging themes and presented these in a summary table with verbatim extracts. Themes were critically discussed with co-investigators (AR, AS DF, NH).
4. The summary table was subsequently presented to the Study Steering Group.

The injury severity score was used to categorise the injury severity. The Injury Severity scale is a retrospective tool used in a clinical setting where injury severity is rated using a numerical scale. The score ranges from 0–75 with a higher ISS score correlated with a greater risk of mortality. 0–8 is characterised as mild injury severity, 9–15 moderate and 10–24 major severity. A score over 25 indicates a profound injury. [37,38].

#### Physiotherapist focus groups.

Analysis consisted of four stages following guidance of the Kreuger Framework: [22,39]

1. NM read the transcripts several times
2. NM constructed a preliminary framework of themes and subthemes supported by supporting verbatim extracts and discussed with focus group moderator (NH)
3. Once the framework was developed, data was indexed and charted using a process of sorting and arranging quotations.
4. The framework was then presented to study management team (AS, AR, DF, NH) and Study Steering Group where the concept was defined, and findings explored for explanations and associations.

### Transparency and trustworthiness of findings

To ensure trustworthiness multiple strategies were employed. Blind reviewing of the data in stage 1 and 2 (NM, MM) was completed. Presentation of the themes both at an early stage and later stage of analysis to the Study Management Group and to the Study Steering Group (NM, PPI co-investigator, LS, AS and independent chair) allowed peer and patient critique and collaborative approach. Additionally, acknowledging lead researchers potential preconceptions and beliefs and encouraging reflexivity enabled transparency [35].

### Patient and public involvement

Patient and public involvement (PPI) has been integral to this project from inception. The study idea was presented to PPI groups at an early stage and has informed the development of the study including giving feedback on the initial idea, time points used and recruitment strategy. Our PPI co-investigator has been part of the study from inception and has provided feedback at the time of protocol and topic guide development and continued participation in the Study Steering Group Committee which included interpretation of the findings. Further detail on the role of PPI and Study Steering Group can be found in the published protocol [20]

## Results

### Participants

#### Patient interviews.

Twenty-five participants were recruited and consented to participate in the study. Of the 25, 8 participants did not complete the interview: 4 participants did not respond to emails/phone calls to arrange the interview, 2 requested to withdraw due to feeling they would not be able to discuss the accident and feeling overwhelmed following discharge, 1 died in the period from recruitment to organising the interview, and 1 experienced a change in mental capacity from recruitment to interview. Therefore, 17 participants were interviewed with their characteristics summarised in Table 1. The average time from injury to completing the interview was 23 days (range 5–44 days). Whilst the majority (n = 12) of the interviews were completed within the 4 weeks of injury. It was not possible due to other medical commitments, e.g., surgery and rehabilitation and being medically unwell to capture all participants within that time period. It was deemed valuable to include the data within the study and this highlights the challenges in capturing experiences at this crucial timepoint. The interviews lasted between 40–60 minutes. The majority of interviews were conducted after the patient had been discharged (n = 13) with n = 4 completed whilst the participant was an inpatient.

**Table 1:** Characteristics for Patient Interviews.

Age Mean (SD)	53.94 (17.16)
Male/Female	Male n = 11/Female n = 6
Mechanism of Injury	Road Traffic Collision n = 8 Fall more than 2m n = 3 Fall less than 2m n = 3 Sporting Injury (fall off motorbike) n = 2 Crush Injury n = 1
Multiple vs Single Injuries	Multiple Injuries n = 11 Single Injuries n = 6
Injury Severity Score	Mild n = 5 Moderate n = 10 Major n = 2

SD – Standard Deviation

#### Physiotherapist focus groups.

Ten physiotherapists (5 per focus group) were recruited and consented (female n = 8, male n = 2). Years qualified ranged from 14 months to 16 years including a range of junior staff to clinical specialist/team leader included. At the time of recruitment, the aim was to recruit both inpatient and outpatient physiotherapists for the focus groups. However, due to staffing and hospital pressures at the time of recruitment, all the participants were currently working within an inpatient setting and no current outpatient physiotherapists were included in the focus groups. This was discussed within the SSG and SMG with the pragmatic decision to stop recruiting for the focus groups. Both focus groups were 60 minutes in duration.

### Findings - emerging themes

#### Patient perspectives.

**Fig 1:**
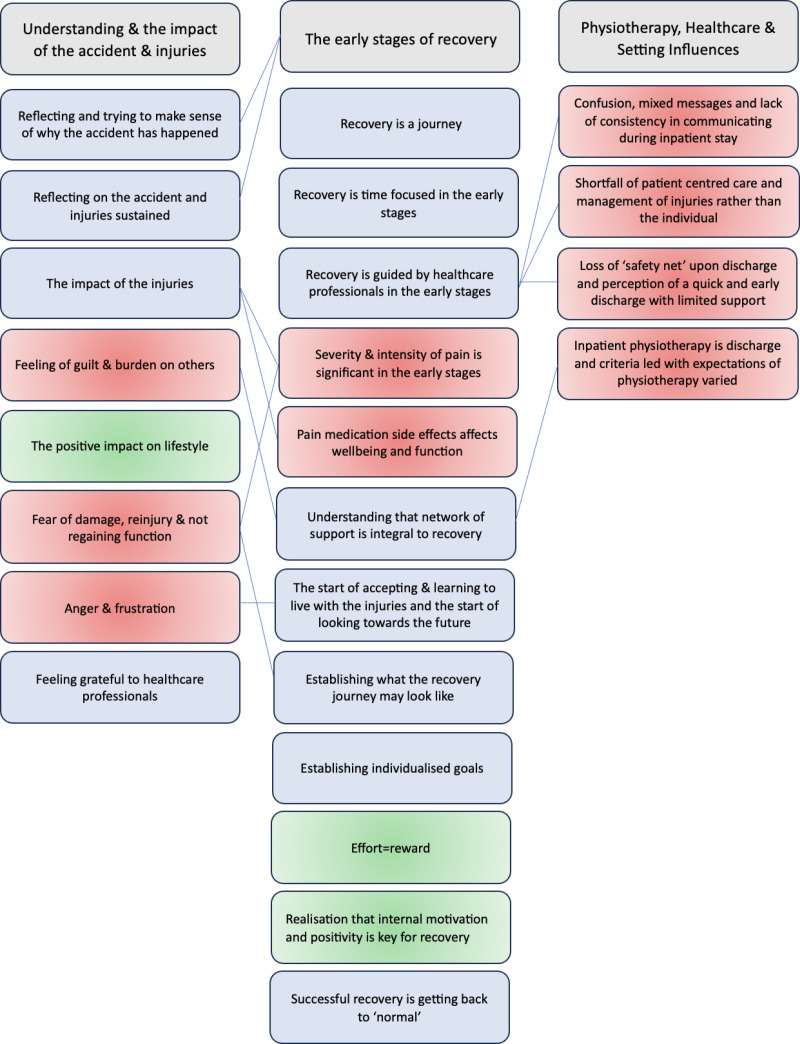
Themes and subthemes of the patient perspectives. Those in blue represent the process of recovery with the red and green highlighting factors which are the researcher’s interpretation of what could negatively or positively impact on the recovery journey.

Three main themes emerged: Understanding and impact of the accident and injuries, the early stages of recovery and physiotherapy, healthcare and setting influences. All participants embarked on a journey of recovery following their injuries and their progression through different stages of this recovery journey was individual regardless of severity of injury, mechanism of injury, age or gender. Some subthemes across the main themes are identified are the ‘process’ in which the participants go through as part of their journey, whereas some subthemes could potentially either negatively or positively impact on their journey and experience of recovery. All themes and subthemes are presented in Fig 1 and Tables 2–4-4.

#### Understanding and impact of the accident and injuries.

Participants discussed the accident and injuries in detail during the interviews and it was clear that processing what happened to them required a significant amount of mental energy. This then linked to theme 2 (the early stages of the recovery journey) where they were starting to process their recovery. The consequences of the injuries affected all aspects of life which linked to the feeling of burden and guilt to which it has impacted on family and others. The loss of independence was evident, but this was not just described as the physical aspects such as walking and activities of daily living, but social aspects being important to them as well. Multiple emotions were highlighted including anger, frustration, and fear. There were however some positive changes to attitude and lifestyle, e.g., quitting smoking and feeling grateful towards healthcare professionals despite their experiences not always being positive (theme 3). Further detail on subthemes can be found in Table 2.

**Table 2:** Subthemes for Understanding and Impact of the Accident and Injuries theme.

Subtheme	Description	Quotation
Reflecting and trying to make sense of why the accident has happened	The majority of participants recalled the accident and their feelings at the time whilst trying to make sense of why the accident happened. Some participants used fault to make sense of why the accident happened whilst others used the conditions at the time to try and make sense of why the accident had happened.	“I’ve done this ride for well over a year, about three/four times a week, probably more than that. A road I know very, very well. Had left in the morning, it was sort of a normal autumn morning. Traffic was a little bit heavier than normal, but nothing out of the ordinary” **Participant 001** *[Male, 33, Multiple Injuries, Moderate Injury Severity]* “So the incident is my fault so that’s why I feel a little bit embarrassed about it. But arguably it could have happened to anybody. You know you’ve got a bend just coming up, if there’s something behind that bend and you don’t know it’s there, I don’t know….. Because I am actually a professional driver you know what I mean. So people are going to really dig at me for this. So I’d rather they didn’t know.” **Participant 020** *[Male, 56, Multiple Injuries, Mild Injury Severity]*
Reflecting on the accident and injuries sustained	The majority of participants discussed the injuries sustained; these were reflected upon in 3 stages: Immediately post injury Operation and initial management Post operatively	Immediatley post injury - “I think I went to grab my legs because they were obviously hurting quite a bit. And this left leg I knew was wrong, because I went to grab it and it just sort of dangled in my hands” **Participant 007** *[Male, 56, Single Injury, Major Injury Severity]* Operation and initial management - “In a nutshell, I was completely taken to pieces, literally to the bones and put back as best they can with some natural things that were there and strong things are now missing” **Participant 008** *[Male, 58, Single Injury, Moderate Injury Severity]* Post operatively - “And I’m concerned about my foot at the moment, I don’t know if I’ve got any long-lasting nerve damage, but only time will tell on that apparently as well” **Participant 010** *[Male, 43, Multiple Injuries, Moderate Injury Severity]*
The impact of the injuries	All participants discussed the impact of injuries with multiple consequences following injury which affected all aspects of their lives and others: Loss of independence Loss of social independence Loss of concentration Impact on work Financial implications Impact on others	Loss of independence - “I can’t go to the toilet on my own, I haven’t been able to go to the toilet, I have to use a bedpan. They won’t let me get on the wheelchair, because they’re worried about me damaging the skin graft and my muscle graft….. Oh yeah, I’m just restricted to being in my bed now constantly since the accident” **Participant 015** *[Male, 35, Multiple Injuries, Moderate Injury Severity]* loss of social independence - “leading a completely normal life and doing all my things at my groups and everything I belong to and all that sort of stuff. And it just stopped. And this isn’t a good a good age to just stop doing things” **Participant 019** *[Female, 76, Single Injury, Moderate Injury Severity]* Loss of concentration - “Initially terrible. I was noticing when I was writing a text message or anything like that, I couldn’t write a sentence. I couldn’t write anything like that and it was getting to the point, what I was doing was just doing voice to text. That was purely all I was doing. I couldn’t write a text message” **Participant 001** *[Male, 33, Multiple Injuries, Moderate Injury Severity]* Impact on work - “I’m off work; they won’t let me work, even from home” **Participant 004** *[Female, 34, Single Injury, Moderate Injury Severity]* Finanial implications - “I’ve lost all my contact numbers, I haven’t got my laptop, I can’t pay my bills, but they won’t be paid. I’ve made a couple of phone calls and said, “Look, I can’t pay because I’m in hospital.” They said, “That’s fine.” **Participant 006** *[Male, 63, Multiple Injuries, Moderate Injury Severity]* Impact on others - “It’s impacted her (daughter) day-to-day life with me, you know, I’ve had to ask her to do everything, including helping me bath and doing stuff that I can’t do with” **Participant 009** *[Female, 44, Multiple Injuries, Mild Injury Severity]*
Feeling of guilt & burden on others	Many of participants described the feeling of guilt in different aspects of life, such as not being able to work, as well as feeling a burden to others around them due to dependence following injury	So I feel guilt, in that regard (not being at work), so yeah, it doesn’t sit easy. But, yeah, that’s how I feel. **Participant 010** *[Male, 43, Multiple Injuries, Moderate Injury Severity]* “I think I just felt like a real burden. And everyone kept saying to me, “You’re not a burden.” Which I know I’m not, but you know, but it was how I was feeling (sniffs). Sorry.” **Participant 009** *[Female, 44, Multiple Injuries, Mild Injury Severity]*
The positive impact on lifestyle	Many of participants discussed how they had experienced a change in attitudes towards a healthier lifestyle as well as how this can aid recovery.	“So straight away, I thought to myself, well tell you what, I’m going to knock that on the head because if smoking is not good for skin regeneration, what’s the point in me doing it?” **Participant 007** *[Male, 56, Single Injury, Major Injury Severity]* “Yeah I mean it’s just a complete life changing experience, completely you know. I mean I just feel lucky to be alive you know and its completely changed my life, my attitude, you know I just want to look after myself more. I’ve lost, my weight is down, I’ve never been so… my weight has never been so low. I mean I’m you know, got down to a really healthy weight now. I just want to look after myself because I’m just so grateful that I’ve got a chance to live, I’m so grateful that I just want to look after what I’ve got left” **Participant 020** *[Male, 56, Multiple Injuries, Mild Injury Severity]*
Fear of damage, reinjury & not regaining function	The majority of participants expressed a fear of damaging or reinjuring themselves and the impact this would have on their recovery. Female participants in particular discussed not regaining full function compared to male participants	“And just fearful of re-dislocation really, which I appreciate once you’ve dislocated it once, there’s a stronger possibility of it happening again, so I get that, yeah” **Participant 010** *[Male, 43, Multiple Injuries, Moderate Injury Severity]* “concerns that it might be really bad and that I might not be able to get anywhere close to my pre-accident function” **Participant 024** *[Female, 43, Multiple Injuries, Major Injury Severity]*
Anger & Frustration	Many of the participants reporting feeling anger, which was directed either internally or externally, depending on whether they felt the blame for the accident lay with themselves or someone else. Frustration was linked to the impact of the injuries and injuries sustained. Feelings of anger and frustration were individual to the circumstances of accident and injuries sustained.	Anger - “It just makes you more angry about the accident, really. It shouldn’t have happened” **Participant 003** *[Male, 71, Multiple Injuries, Mild Injury Severity]* Anger - “I think I was just annoyed with myself that... and I don’t think I realised at that point the implications of getting myself back on my feet” **Participant 009** *[Female, 44, Multiple Injuries, Mild Injury Severity]* Frustration - “I do still get a little bit frustrated with it (the injuries) you know. It’s a bit frustrating sometimes” **Participant 020** *[Male, 56, Multiple Injuries, Mild Injury Severity]* Frustration - “But I do feel, considering that I’m sitting around doing nothing all day, I do feel pretty tired still, which I find frustrating. But I gather it’s not unusual” (Laughs) **Participant 024** *[Female, 43, Multiple Injuries, Major Injury Severity]*
Feeling grateful to healthcare professionals	Many of the participants commented on the care received; some were more generic about the NHS but others discussed specific individuals involved in their care.	“So yeah, when it comes to NHS, and I’d never been an NHS basher in the slightest, I never realised about what the NHS can absolutely get done, excuse me……. Never ever once realised as to how much resource the NHS can pull together instantly” **Participant 008** [Male, 58, Single Injury, Moderate Injury Severity] “So the physios were great, everyone was great, I can’t praise them high enough at the hospital, it was really good” **Participant 001** [Male, 33, Multiple Injuries, Moderate Injury Severity]

NHS – National Health Service

#### The early stages of the recovery journey.

All participants embarked on a journey following injury but the speed at which they progressed through recovery was individual and appeared not to be related to injury severity, physical injury healing times, or time elapsed since injury. For example, a participant may have a more established understanding of what their recovery journey and successful recovery may look like. Other participants may be unsure what their recovery may look like and are looking for more guidance from healthcare professionals to help understand this journey and therefore have less focus on what successful recovery may look like. There were multiple reasons why some participants had a better understanding of their recovery journey, and this was individual to them. All subthemes in the early stages of the recovery journey give context for their current definition of successful recovery with successful recovery defined by all patients as ‘returning to normal’. This definition of normal was individual to them. Further detail on subthemes can be found in Table 3.

**Table 3:** Subthemes for the early stages of the recovery journey theme.

Subtheme	Description	Quotation
Recovery is a journey	The majority of participants describe recovery as process and is closely linked to getting back to pre-injury function/level.	“I guess I would have seen it as a process of trying to get back to as close as to what I’d be before an injury. ……It’s a sort of linear process of trying to get back to as close to the point you were at before really” **Participant 024** *[Female, 43, Multiple Injuries, Major Injury Severity]*
Recovery is time focused in the early stages	The majority participants highlighted a time focus to recovery which was specific and related to healing times of physical injuries which was communicated to them by healthcare professionals. Some were more generic, and function focused which were of a longer time period	“We were given six to eight weeks for the bones to knit and probably three to four months before I’ll be riding a bike again. So they’re the things I’m focusing on” **Participant 003** [Male, 71, Multiple Injuries, Mild Injury Severity] “I mean they told me I’d be like that for at least 12 weeks maybe 18 weeks before that swelling starts to reduce” **Participant 020** [Male, 56, Multiple Injuries, Mild Injury Severity] “And I’ve had a few physios saying that I’m not going to be able to walk for the next, like, six months to maybe a year properly. So, they’ve told me to rule those jobs out, unfortunately, so yeah” ***Participant 015*** *[Male, 35, Multiple Injuries, Moderate Injury Severity]* “And I mean, at hospital, I was told four to six months before I’m at contact sport, and then nine months for my bone to fully heal” **Participant 018** *[Female, 19, Single Injury, Moderate Injury Severity]*
Recovery is guided by healthcare professionals in the early stages	The majority of participants highlighted the need for guidance by healthcare professionals at this stage during recovery. This subtheme is closely linked to time focus subtheme and participants use this guidance to make sense of what their recovery journey may look like as they seek to gain an understanding	“With this medical stuff, I’m prepared to be told, “This, that, do that, do that.” I leave it totally up to them, because they obviously know what they’re talking about, and they’ve spent a lot of time studying and passing exams to do that”….. And as long as they keep pointing me in the right direction and telling me what to do, then I’m happy about that because I don’t know a lot about it” **Participant 007** *[Male, 56, Single Injury, Major Injury Severity]*
Severity & intensity of pain is significant in the early stages	Thirteen participants discussed how pain was a significant experience both at the time of injury and during the early stages of recovery. This affected all aspects of functioning and impacted on their ability to participate in rehabilitation.	“they got me out once to try and get me walking, but it was just, the pain was just unbelievable. I literally shuffled to the end of the bed and back. It was just too much” **Participant 003** *[Male, 71, Multiple Injuries, Mild Injury Severity]* “Every time I tensed my leg, it was so painful. It was just another type of pain; it was horrible” **Participant 018** *[Female, 19, Single Injury, Moderate Injury Severity]* “which I could tell you about them because they (ribs) hurt. One is bad enough, but four, oh, God, that really isn’t funny…… if I go to bed, I wake up and I’m in agony in the chest….. My wrist is exceedingly uncomfortable” **Participant 021** *[Male, 66, Multiple Injuries, Moderate Injury Severity]*
Pain medication side effects affects wellbeing and function	Several participants highlighted a range of side effects which ranged from mild to more severe.	“the pain medication needed to come out of my system because I really did not feel good on it at all. And it was actually hindering my rehab as well, because I couldn’t really sit up for long periods of time without really my head hurting or spinning a lot” **Participant 004** *[Female, 34, Single Injury, Moderate Injury Severity]* “I will say I was pretty poorly with the medication as well, so I was taking morphine and it was making me poorly. Very, bad hallucinations, very suicidal thoughts at night. I thought I was having a dream where I was suffocating, and I woke up as if I was. Yeah, all sorts of nasty, nasty things” **Participant 010** *[Male, 43, Multiple Injuries, Moderate Injury Severity]*
Understanding that network of support is integral to recovery	The majority of participants were appreciative of support they had received from family and healthcare professionals. Support was integral to recovery with plans on how this will integrate into recovery discussed.	“I’m really, really lucky that I’ve got a really lovely girlfriend who is supporting me, who is able to do a lot of that for me. If I didn’t, I’d be stuck. I live in a one bed; I live with a landlord” **Participant 001** *[Male, 33, Multiple Injuries, Moderate Injury Severity]* “I feel fairly positive because I do have a good support network in my family…..But I can imagine feeling very, very anxious… and feeling like there would be a lot of barriers if I did not have that external ability to help myself” **Participant 024** *[Female, 43, Multiple Injuries, Major Injury Severity]* “I think regular support from the physio. My wife, my wife will be very pushy, once she knows, because she’s, let’s say, of a caring nature, yeah….. And I’m very fortunate” **Participant 010** *[Male, 43, Multiple Injuries, Moderate Injury Severity]*
The start of accepting & learning to live with the injuries and the start of looking towards the future	The majority of participants had started to process the acceptance of injuries. This was not related to injury severity and appeared not to be related to whether they expected to make a full recovery from their injuries. Participants were at different stages of acceptance. Two distinct phases were identified: Learning to accept and live with the injuries Looking towards the future and what this might look like	Learning to accept - “I’ve accepted the injuries because I can’t do anything but, and obviously I want to get well again” **Participant 006** [Male, 63, Multiple Injuries, Moderate Injury Severity] Learning to accept - “It’s just to coming to terms with the timescale of these injuries. It’s a slow recovery process and I still haven’t completely come to terms with that….. Well I don’t know, I suppose just learning to live with these injuries really. There’s nothing I can do about it.” **Participant 020** *[Male, 56, Multiple Injuries, Mild Injury Severity]* Looking forwards - “But yeah, if I can do as much as I can, then I’ll just have to tailor stuff after that, so get back to work, get back to living a normal life, really.” **Participant 007** *[Male, 56, Single Injury, Major Injury Severity]* Looking forwards - “No, you know, I’ve kind of accepted the fact it’s happened. The only thing I can do now is just get better as quick as I can do and recover as quick as I can do. And I know that my foot’s never going to be 100% the same” ***Participant 015*** *[Male, 35, Multiple Injuries, Moderate Injury Severity]*
Establishing what the recovery journey may look like	The majority of participants had started to process what recovery may look like and this was individual and varied to what stage of the recovery journey the participant was in at the time of the interview. Some distinct similarities to help the participants understand their recovery journey were notable: Establishing the possibilities of recovery Acknowledging uncertainty Positivity	“Initially I was like, a couple of weeks and I’ll be back to normal. And now it’s gone from a couple of weeks to I’m… if I can walk before Christmas I’ll be laughing” **Participant 001** *[Male, 33, Multiple Injuries, Moderate Injury Severity]* “So I haven’t got a clue how that’s (hobbies) going to pan out. I’m hopeful that we’ll get somewhere with it. But yeah, that one is on the ‘maybe’ list still. And it’s definitely on the maybe should, rather than maybe shouldn’t” **Participant 008** *[Male, 58, Single Injury, Moderate Injury Severity]* “I feel positive about getting where I need to get to, it’s just the time it’s going to take to get there” **Participant 010** *[Male, 43, Multiple Injuries, Moderate Injury Severity]*
Establishing individualised goals	All participants discussed goals in their recovery journey with some setting multiple goals. Goals were varied and individual to the participant. Goals were focused on physical aspects such as walking and regaining independence with some participants focused on social aspects and returning to work.	Well, just to be able to actually put my weight on it again. **Participant 014** *[Female, 72, Multiple Injuries, Mild Injury Severity]* “But for me, I think, if I can get some movement, some decent grip in my hand and be able to scratch right ear with my right hand and eat my dinner with a knife and fork and drive, then I’d say I’ve done all right.” **Participant 008** *[Male, 58, Single Injury, Moderate Injury Severity]* “My medium-term goals are to be able to be back caring for the kids and doing school runs and driving and things. And longer than that would be back to be able to do… I normally have a whole load of bird surveys that I do in April/May time” **Participant 024** *[Female, 43, Multiple Injuries, Major Injury Severity]* “I suppose the short-term goals will be getting back to more of a normal life. Getting out and about, seeing people, maybe nipping over the pub now and again to see people because they’ve all been very good over there” **Participant 007** *[Male, 56, Single Injury, Major Injury Severity]* “I would love to go back, it’s not a job that I do because I have to do it but I like it” **Participant 012** *[Male, 78, Multiple Injuries, Mild Injury Severity]*
Effort = reward	Many of the participants discussed their perception that putting more time and effort in was directly related to getting more out of recovery overall.	“But in the back of my mind, it’s like well, if I put 200%, a bit more effort in, the chance that I could get it stronger than before. I could build upon it, build the muscle within my leg stronger than ever before, then that might be more beneficial to me than it was before” **Participant 015** [Male, 35, Multiple Injuries, Moderate Injury Severity] “I put in the effort, so I was, you know… it felt more rewarding. So I’m thinking my leg will be the same, once I get through it. Like I said, little wins make a big, big difference at the end of the day.” **Participant 018** *[Female, 19, Single Injury, Moderate Injury Severity]*
Realisation that internal motivation and positivity is key for recovery	The majority of participants discussed the concept of internal motivation and that being positive is important for recovery.	“I’ve just got to get better, you know, determined that I’m going to push through it” **Participant 012** *[Male, 78, Multiple Injuries, Mild Injury Severity]* “If I’ve got a good mindset, and I’m doing my exercises and I’m doing everything I’m told, in a way, as frequently as I can, it will help” **Participant 018** *[Female, 19, Single Injury, Moderate Injury Severity]*
Successful recovery is getting back to ‘normal’	All participants described successful recovery as getting back to ‘normal’. The definition of ‘normal’ was individual, with some participants giving specific examples whilst others being more generic to lifestyle. This was not related to injury severity, number of injuries, age or gender and the pre-injury level was frequently referred to.	“Like, when I am back to my normal self, doing my normal activities, no crutches, no nothing, I will be recovered…… yeah. I mean, like, 100% physical normality; like, back to my exercise regime, full social thing, back to work – like, back to normal.” **Participant 004** [Female, 34, Single Injury, Moderate Injury Severity] “I want to be able to go back to what I was doing before.” **Participant 006** [Male, 63, Multiple Injuries, Moderate Injury Severity] “Successful would be as I was before, as simple as that. 100% successful is no change, no limitations” **Participant 010** [Male, 43, Multiple Injuries, Moderate Injury Severity] “Yeah, if I’m restricted in any way because of what’s happened, then I would say I’ve not had a successful recovery” **Participant 003** [Male, 71, Multiple Injuries, Mild Injury Severity] “It would just be normal, as in as close to what I had had before, mobility usage…. Normal would be how I was before and acceptable would be doing the things I did before, but perhaps with a slightly different, maybe not being able to do things quite as fast or as easily or perhaps with some level of discomfort. But being able to do them” **Participant 024** *[Female, 43, Multiple Injuries, Major Injury Severity]*

#### Physiotherapy, healthcare and system influences to recovery.

There was a clear focus on the management of injuries rather than the individual with patient centred care often being overlooked. Discharge was often seen as being rushed, disjointed, and criteria based rather than patient led. For those who had been discharged at the time of interview, there was a feeling of a loss of a safety net upon discharge which created anxiety and worry amongst some participants and a heavy reliance on their support network (theme 1). There was a feeling of mixed messages with lack of consistency in what patients were told during their inpatient stay which impacted on their understanding of their recovery journey. Further detail on subthemes can be found in Table 4.

**Table 4:** Subthemes for Physiotherapy, Healthcare and system influences to recovery.

Subtheme	Description	Quotation
Confusion, mixed messages and lack of consistency in communicating during inpatient stay	Many of the participants reported negative experiences, particularly around communication, and the feeling of confusion as a consequence. There were some positive experiences expressed by six participants, with four participants discussing both positive and negative experiences during their recovery so far.	“I had a number of different departments, all these people coming round and telling me this, telling that, and I wasn’t of a clear mind to rationally think about what the hell was going on” **Participant 010** *[Male, 43, Multiple Injuries, Moderate Injury Severity]* There were people… the anaesthesiologists came and saw me and said, “You’ll be in the hospital for a week.” The consultant told me, “You’ll be here for three or four days.” And then the physio, one day post op, was like, “Oh, we’re going to discharge you tomorrow.” **Participant 004** *[Female, 34, Single Injury, Moderate Injury Severity]* “Everybody here knew, I didn’t know. It just would be nice just to say, “Right, this is what’s going to happen.” **Participant 017** *[Male, 59, Multiple Injuries, Moderate Injury Severity]* “And I think everything that they (physio) say makes a lot of sense, like, in terms of the phases that I’ve gone through. Like, I went through the phase where like the entire injury….. So, I think they’ve been really good about explaining to me the phases that I’m going through” **Participant 004** *[Female, 34, Single Injury, Moderate Injury Severity]*
Shortfall of patient centred care and management of injuries rather than the individual	The majority of participants described the feeling of their injuries being managed rather than a person throughout the early stages of recovery, from being in the ambulance to discharge from hospital.	“But they can’t see you and they can’t hear you but you’re in the corner listening to them, and that’s what it felt like in the ambulance after all that. I thought well, who are all these people? I can hear them all muttering and chattering away about me, but they don’t seem to actually acknowledge that I’m there” **Participant 007** *[Male, 56, Single Injury, Major Injury Severity]* “I was quite upset, because they wouldn’t obviously let me daughter come in the ambulance. Which I was upset that I had to leave her in the middle of nowhere to get herself home” **Participant 009** *[Female, 44, Multiple Injuries, Mild Injury Severity]* “I didn’t wear underwear for three days when I was in Coventry because I didn’t have any and I didn’t have anyone nearby who could bring me anything. There were no visitors, there was nothing, so I didn’t wear underwear for three days. I didn’t have an iPhone charger; I didn’t have anything with me. I didn’t have any shoes because my boot, they took it off in the field, so I had one boot with me. I didn’t have any clothing” **Participant 004** *[Female, 34, Single Injury, Moderate Injury Severity]* “And I can totally understand that because I can see from within, inside the NHS, I could see how utterly stressed it is. There’s a sort of cascade of care from amazing at the critical points to just downwards and outwards to almost nothing at the edges” **Participant 024** *[Female, 43, Multiple Injuries, Major Injury Severity]*
Loss of ‘safety net’ upon discharge and perception of a quick and early discharge with limited support	Many of the participants described a strong feeling of losing a safety net upon discharge. This created some anxiety with some participants questioning how they would cope upon discharge	“you’ve lost your safety net coming out of the hospital and that was a bit of a thought that I was worried about” **Participant 001** *[Male, 33, Multiple Injuries, Moderate Injury Severity]* “I’d have had a nice little call button here, to go bing and they come along and go, “What’s the matter?” And I’d say, “Well, this, that, the other, blah, blah, blah.” But I couldn’t do that, so I just had to deal with it and make the best of it” **Participant 007** *[Male, 56, Single Injury, Major Injury Severity]* “So, then physios came into my bed, just to get me out of bed a bit, which I found quite hard but anyway, we got through it. They got me on crutches and they then said to me, one lunchtime, “Would you be prepared to have a go at getting up the stairs?” I said, “Yes, no problem.” So, I managed to get up the stairs and back down the stairs no problem and they said, “Well, you don’t need to go to hospital, you need to go home. There’s nothing wrong with you if you can get up the stairs at home, you can go home.” **Participant 012** *[Male, 78, Multiple Injuries, Mild Injury Severity]*
Inpatient Physiotherapy is discharge and criteria led with expectations of physiotherapy varied	Physiotherapy was described by the majority of participants with the discharge process described as criteria led such as being able to complete the stairs. The expectation of physiotherapy varied, and this affected satisfaction levels.	“I mean they had criteria for when you should be discharged. I was washing myself and dressing and I could transfer from the bed to the chair and sit. So as far as they were concerned, I was ready for discharge” **Participant 003** [Male, 71, Multiple Injuries, Mild Injury Severity] “Actually on the day I was discharged they took me, two physiotherapists they took me to a room and got me to go up some steps, low steps and stuff like that. So they were happy that I could use the crutches safely” **Participant 020** *[Male, 56, Multiple Injuries, Mild Injury Severity]* “So, the physios came and visited twice, but, you know, the extent of what we did is we kind of had me walking to the bathroom and that was all we did” **Participant 004** *[Female, 34, Single Injury, Moderate Injury Severity]* I don’t know. I found the physio really good. Even on the ward, I found it really good. Both, I had two ladies on the ward….. and they’ve been really great. And I think I’m one of these that I’ll automatically say, “Oh, I can’t do this.” And especially when I was on the ward, I was literally... they’d say, “Right, you’ve got to do this…… I think they’ve been really instrumental in making me believe that, come on, you can do it, you know” **Participant 009** *[Female, 44, Multiple Injuries, Mild Injury Severity]*

#### Physiotherapist perspectives.

**Fig 2:**
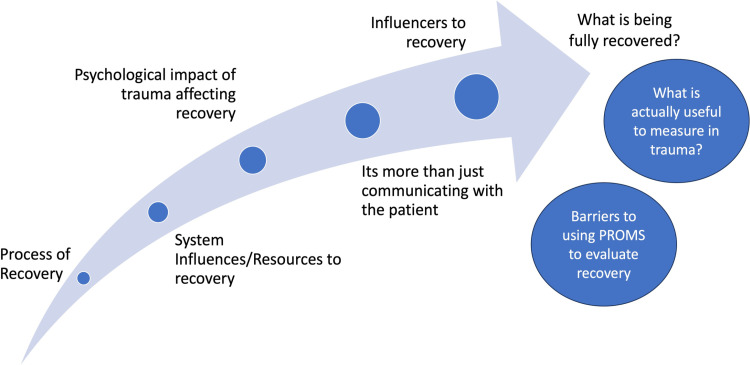
Themes of physiotherapist focus groups demonstrating the different themes which could contribute to being fully recovered along this journey.

Eight overarching themes were identified: Process of recovery; What is being fully recovered?; It’s more than just communicating with the patient; Psychological impact of trauma affecting recovery; System influences/resources for recovery; Influencers to recovery; Barriers to using patient reported outcome measures (PROMs) to evaluate recovery; What actually is useful to measure in trauma? All themes and subthemes are summarised in Fig 2 and Table 5

**Table 5:** Subthemes for all themes for the focus groups.

Subtheme	Description	Quotation
**Process of Recovery**		
Recovery is a journey	There was a description of a journey following injury with some feeling there is an endpoint to recovery where others felt was more an everlasting journey.	“I don’t tend to see it as a final state, it’s a kind of a transient state which you keep on going through until you reach that endpoint” “Recovery is like an everlasting thing, a journey rather than just one single… you’ve met that and you’ve recovered, off you go”
Recovery has to be meaningful and individual	There was agreement that recovery is individual and to what the patient wants to achieve.	“Sometimes it’s a lot of what a patient defines as recovery rather than what we would define”
Gaining insight of the injuries is part of the recovery process	There was the feeling that in the early stages of recovery that patients’ insight into their injuries can influence their expectations and that this insight takes time to understand.	“I think their expectation of what they will achieve is probably lower than what it would it be further along the journey……But actually once they start to visualise that recovery beyond that is possible, I think perception changes”
Education is an important part of recovery	Gaining an insight was closely linked to education being an important part of recovery journey with physiotherapists being integral to this.	“I think we educate and re-educate. Like often patients are bombarded with a lot of information in that really acute phase and it’s very difficult for them to properly take in and digest……, while they’re here they get comfortable to ask us questions, asking to explain things when they don’t understand”
**What is being fully recovered?**		
When the patient is happy with their current level of function	There was emphasis that being fully recovered is individual to the patient and is when the patient is happy with their level of function.	Personally, I think it’s when a patient accepts that or feels happy and content, and feels that they can manage at that level”
When recovery is meaningful and important to the patient	It was acknowledged that what was important did vary between patients and this could then affect what was important to patients in terms of being fully recovered.	“And usually it’s, like what you’ve said, it’s being able to get back to what makes their life most meaningful and most fulfilling. So, for someone our age it might be getting back to sport that they played previously, or being able to do their job comfortably. For elderly people it might be able to walk on not very solid ground without falling over”
Returning to or near a preinjury level of function	There was agreement that returning to preinjury level and good functional level was important to patients following injury.	“A return to a reasonable quality of life…….… injury, function, baseline”
**It’s more than just communicating with the patient**		
Communication within and between MDT members and patient is key	There was emphasis on communication and how poor communication can impact recovery journey experience for patients. The communication across all members of multi-disciplinary team and within speciality was important to avoid miscommunication and consistent messaging.	“Yeah, I think it is that care in-between as well…. and like communication across the whole of MDT as well. So, not getting mixed signals, mixed information. And making sure that there is a standardised front going forward on what we’re doing with the patient”
Active listening to the patient and being involved in decision making	The patient voice was highlighted as being important in the recovery journey, but this was not extended to decision making of recovery in discussions.	“Yeah. That they’re listened to… And not just told what they’re going to be doing over their rehab”
**Psychological impact of trauma affecting recovery**		
Every survivor of trauma is impacted psychologically	There was a sense that you didn’t need to have a ‘diagnosis’ of psychological difficulties in medical terms but there will be an impact psychologically in some sense and this can impact recovery.	“Psychological impact is more the emotional wellbeing of that individual, how they respond psychologically to it and the impact that that’s having on their wellbeing and their recovery”
Loss of independence and identity experienced	Loss of independence was highlighted and how this can impact on a person psychologically.	“I guess a lot of those things we’re talking about is in terms of work and hobbies are the things that make up a person as well. So, if you were a person that was previously able to do these things and now you’re not, it’s kind of like a loss of identity and alongside the psychological impact of that”
Period of adjustment needed for journey transitions	The early stages following injury were discussed as being challenging for the patient, and there is a lot to process which was linked to psychological wellbeing.	“I think in the initial stages they have so much going on; they’re trying to come to terms with the incident, the injuries that have happened, that it can all get a bit overwhelming, and they don’t necessarily have the insight of the psychological impact that is having on their recovery and their rehab”
Fear about getting back to ‘normal’	Negative emotions in particular fear was emphasised, highlighting the ‘unknown’ at the early stages of recovery.	“I suppose the fear about getting back to normal, are they ever going to get back there. Are they ever going to be able to work again”
Acceptance of recovery journey challenging	Acknowledgement that some patients take time to process recovery and the difficulty accepting how long recovery takes was discussed.	“I think it sometimes difficult for some individuals to come to terms with the length of that recovery”
**System Influences/Resources for recovery**		
Hospital pressures impacts patient journey	It was highlighted that hospital pressures affect how patients are managed, consequently patients are not at the centre of their care.	“I think we get focused very much on what our goals are……it’s very easy at the moment because there’s such pressures on in the hospital to get in, to get out, to get the next bed because there isn’t much of them about, which I know I don’t think of the recovery pathway as like what the patient wants as much. It’s literally, “Can we get this patient out? Are they at a point where they’re safe to go to some sort of facility or to go home?” And then that’s our bit done”
Lack of long-term provision/rehabilitation effect on patient journey to recovery	It was highlighted that there was a need for a specialist rehabilitation facility upon discharge, but this currently is not available within the NHS for musculoskeletal partients.	“NHS is lacking having actual specific rehab facility or provision for the polytrauma patients. So, like if they’ve been more complex patients with a higher severity injury, you’ve got it for your neuro rehabs, you’ve got it for your spinal rehabs and all of that. But there’s nothing for the MSK, especially if you’re under 65. And, to be fair, that’s the group we really should be focusing on because they’re the ones that are going to be returning to work and they’re going to have a bigger effect on the economy for the payments going forward, with the care and everything from that point of view as well”
System drivers are not enabling a focus on psychological support in the acute phase	There was discussion around specific training need for psychological support for physiotherapists but then a wider discussion around the system following discharge doesn’t enable adequate support in the community.	“I think really in the way that we are trained, we are really poorly equipped to support patients from a psychological point of view….. But in terms of empowering them to want to do more we aren’t really educated on how to do that” “So, yeah, it is lacking, and I definitely think even when it goes to be discharged as well, the psychological support in the community isn’t necessarily there for them either”
System changes to psychological support beneficial	It was acknowledged that there was recent change within the service with addition of psychological support, and this was not only benefiting patients but also the relationship between MDT members.	“Fortunately, here we do have access to an assistant psychologist who we can refer to if we think engagement is becoming an issue with therapy, but I think if you’re then out in the community or you’re out in MSK services and you’re experiencing that same drop in engagement, we’re not really empowered to provide that for patients” “I think it’s opened channels of communication, hasn’t it? More so I suppose that we wouldn’t have previously had I would say as the same lady, the assistant psychologist, she has… we’ve asked her to review a patient who we felt was quite low in mood and she would do it for us and then she on two or three occasions has sent back to me about how best to order, how to approach this patient and get more kind of participation”
**Influencers to Recovery**		
Finance and occupational pressures can influence recovery	It was highlighted that external factors can influence recovery with a particular focus around returning to work or not being able to work and financial pressures.	“Yeah and definitely those external factors like you say, the money aspect, the job aspect and things it can definitely play a part in their recovery and kind of the way they go”
Psychological wellbeing influences motivation and participation in rehabilitation	There was a sense that participation was directly influenced by psychological wellbeing emphasising the importance of a holistic individual approach to recovery.	“It’s a big factor towards the patient’s motivation in participation because if we have any patients who are having kind of flashbacks or nightmares or are low in mood secondary to their trauma it decreases the likelihood of participation, unfortunately”
More severe injuries have a longer journey of recovery and influences recovery endpoint	There was a feeling that level of recovery was directly linked to severity of injury and more physical aspects of the injury.	“I think the longer-term disability of the patients who have got more severe injuries, are they likely to have residual loss of function that means that they are less likely to achieve recovery in whatever sense that would be. I think that’s kind of what you would classify as more or less severe, and in that case I’d say your patients are less likely to recover, therefore less likely to reach their goals. Therefore, more likely affected”
Medical management & prioritisation influences recovery journey	How a consultant chooses to manage an injury can be individual to the patient, and is often conflicted with expectations of family members, as well as lifesaving treatment taking precedent over other injuries such as musculoskeletal injuries. All of which can influence the recovery journey.	“So what the consultant wants initially might change and what they’re happy with might change. That might influence that weight bearing status, but family members expect certain things in recovery. Then if patients aren’t doing X, Y, Z that influences the patient” “Say where their priority is to treat their chest to save their lives essentially, they tend to look after their MSK injuries less”
**Barriers to using PROMs to evaluate recovery**		
Impractical to use PROMs in an inpatient setting	There was the impracticality of using PROMs in a clinical setting but also discussion around the perception of not being able to access some outcome measures which may be suitable to due licensing restrictions.	you either make them really long and tedious that not many people will do them. Or you make them really short where they’re not specific” “because we’re only staff, you have to get the, what’s it called, the copyright, the licence, haven’t you?”
Current PROMs lack specificity	The variety in the injuries and patients which the staff see following injury was acknowledged and a factor which contribute to outcome measures not being specific enough to capture change.	“I think outcome measures as much as they are useful they’re never specific enough to use in an acute setting because there’s just such a disparity between the type of patients that you get and what you’re trying to achieve with them”
Clinical practice does not involve routine use of PROMs	There was a sense that outcome measures were only PROMs and these weren’t used routinely but objective outcome measures were used routinely.	“Obviously we use our objective markers which is range and things, but we don’t really use outcome measures” “We’re not particularly good at this level at doing outcome measures”
**What actually is useful to measure in trauma?**		
It is unclear what outcome measures are useful PROMs, physical measures or both?	There was disagreement to what is more suitable to use to measure recovery within and between the two focus groups. Some felt PROMs were suitable where others felt physical measures were more important.	“I think using more patient specific ones, questionnaire, quality of life, EQ5Ds” “We don’t really use anything like lower extremity functional scale or anything like that, which would be useful because that does measure on disability and impairment” “Yeah. The patient reported outcome measures….. You have also got to look at objective measures as well”
Is function best to measure? One size doesn’t fit all	There was no one answer in terms of what should be measured with most agreeing that function is important, but this doesn’t apply to all patients.	“I think that (getting out of bed, mobility) captures one element of that physical journey but certainly it’s only the bigger sort of qualitative, how do you say, quality of life. That’s how you need to capture that across” “Yeah function. The ability to do the stuff they need to do when they initially get home in my case, but down the line all the other things”
What we need to measure is currently unknown	There was a strong feeling that one outcome measure doesn’t exist, and it is unknown what we should be measuring across the journey of recovery.	“So, to be honest I don’t think we necessarily know what we’re even looking at for outcome measures to see if it’s successful or not” “Absolutely no outcome measure that can follow the patient from first time outside to end of recovery, it doesn’t exist”
Diversity of population makes it challenging to capture specifics of ‘recovery’	It is acknowledged that whilst there are similarities in terms of recovery it is individual, so it is difficult to capture this in an outcome measure.	“Because every stage is so different and there’s no way of quantifying the amount of progression that you’ve made in one stage of recovery to another. So, it almost becomes a little bit redundant to have anything objective because nothing could signify that change from one sort of phase to another”

MDT – Multidisciplinary Team, NHS – National Health Service, PROMs – Patient Reported Outcome Measures

#### Process of recovery.

There was agreement that there is a process and stages of recovery that all patients experience, and that recovery is individual to the patient. It was highlighted that how patients understand and process the injuries sustained is part of recovery and education throughout was an important aspect of recovery. Further detail on subthemes can be found in Table 5.

#### What is being fully recovered?.

There were multiple definitions of being fully recovered but importance was given to the idea that being fully recovered is individual to the patient. Recovery was defined as the patient’s pre-injury status but also defined as when the patient is happy with their level of function. However, in theme 6 (influencers to recovery), it was acknowledged that reaching pre-injury status/level of function wasn’t always possible for patients and this was related to injury severity highlighting the difficulty in one clear definition for being fully recovered. Further detail on subthemes can be found in Table 5.

#### It’s more than just communicating with the patient.

Communication was discussed throughout the focus groups as being of high importance. In particular, communication between multidisciplinary team (MDT) members to inform practice and ensure consistent messaging as well as good communication with the patient was emphasised. It was also acknowledged that it’s not just about communicating to the patient but that actively listening to patients is also important in recovery. There was no discussion around active listening to MDT members. Further detail on subthemes can be found in Table 5.

#### Psychological impact of trauma affecting recovery.

Psychological impact was discussed as affecting all who sustained a traumatic injury, but it was acknowledged that this didn’t mean a ‘formal diagnosis’ of a mental health disorder such as PTSD. Loss of independence and identity, fear of not returning to normal, acceptance, and a period of adjustment were all acknowledged as psychological factors observed which can affect the recovery journey. Further detail on subthemes can be found in Table 5.

#### System influences/resources for recovery.

Hospital pressures were discussed at length and how this affected the patient journey and recovery as well as lack of provisions for musculoskeletal trauma participants such as long-term rehabilitation and psychological support. It was highlighted that there was change within the local service to address some of these provisions particularly around psychological intervention and support, and this impacted positively on the recovery journey. Further detail on subthemes can be found in Table 5.

#### Influencers to recovery.

A number of factors physiotherapists have observed which could influence patients’ recovery were discussed, which included financial and occupational pressures, psychological wellbeing, medical management, and severity of injury. Further detail on subthemes can be found in Table 5.

#### Barriers to using patient reported outcome measures.

Multiple barriers were voiced when discussing PROMs including the practicality of using them in an inpatient setting (e.g., too long to complete), lack of specificity in capturing change and progress, and routine clinical practice does not use PROMs. Further detail on subthemes can be found in Table 5.

#### What actually is useful to measure in trauma?.

There was discussion around what would be useful to measure following musculoskeletal trauma and whether PROMs or physical measures would be useful and whether function would be more appropriate to monitor. There was also discussion around whether there is a full understanding of what needs to be measured is currently unknown and that the heterogeneity in the musculoskeletal trauma population makes this challenging. Further detail on subthemes can be found in Table 5.

#### Coherence and differences between patient and physiotherapist perspectives.

Coherence between themes of patients and physiotherapists were identified across all themes. These included that recovery is a journey and this needing to be individual and meaningful to the patient. Both patients and physiotherapists talked about the psychological impact on recovery including fear, adjustment and acceptance following the accident and accepting the injury were important and can affect recovery. It was recognised that patients wanted to focus on positive aspects of recovery and positivity and wellbeing was important for successful recovery. Both patients and physiotherapists discussed a shortfall in care with patients describing their care as being criteria led and discharge being quick and not well supported. Physiotherapists echoed this describing that the hospital system and pressures in the hospital impacts their ability to engage fully with the rehabilitation needs of the patient.

**Fig 3:**
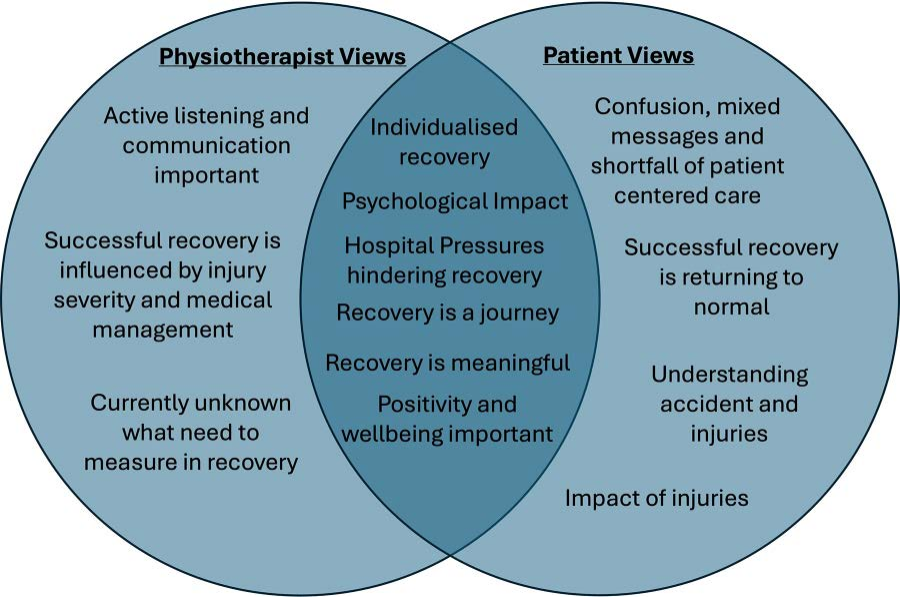
Illustration to demonstrate some of the themes which are similar/different between the patient and physiotherapist findings.

There was, however, differences observed between patient and physiotherapist views. The physiotherapists highlighted that communication, active listening and education was integral to the recovery journey and successful recovery and this was part of routine practice, yet patient views and experiences reported mixed messages and confusion. The definition of recovery also differed with the patients reporting this as normal and as they were before with the physiotherapist’s definition centered around a medical model framework which included to the severity of injury and medical management of the injuries. Finally, the patients discussed in detail trying to make sense of the accident and injuries and the impacts this may have on them, but this was not discussed or highlighted within the physiotherapist focus groups. Fig 3 illustrates the common themes which are similar/different between patients and physiotherapists

## Discussion

This study aimed to explore the patient and physiotherapists views and perceptions of recovery and what constitutes recovery in the early stages of recovery following musculoskeletal trauma. The findings highlight the complexity of recovery in this population.

Key findings from patient interviews indicate that understanding and processing the accident and injuries are significant in the early stages of recovery. The early stages of recovery was similar irrespective of injury severity, age or gender, and did not appear to follow standard soft tissue healing trajectories. Successful recovery was consistently defined as returning to ‘normal’. Multiple influences can positively or negatively affect the early stages of recovery.

Key findings from the physiotherapist focus groups showed similarities to patient interviews where system influences and influencers to recovery were discussed. However, differences in views on communication and definitions of successful recovery were noted. Barriers to using PROMS and what is useful to measure in trauma rehabilitation demonstrated the complexity in this population. Physiotherapists highlighted that recovery was individual but their definition of successful recovery was more to do with when the patient was happy with their level of function. Communication was emphasised as being important with the MDT and the patient, with psychological impact noted as being significant. Other factors influenced recovery from both system and individual perspectives. There were multiple barriers to using outcome measures with no single outcome measure being fit for purpose in this population.

The findings support current literature that the recovery process is complex and individual, with issues of poor communication with healthcare staff and multiple emotions experienced by patients [13,40–45]. The study also adds new insight into the lived experience at at a crucial early timepoint following soon after the injury and accident has occurred. Key new findings include that patients define recovery as returning to ‘normal’ soon after the injury has occurred. Crucially, this differs from the physiotherapist views which is more aligned to a medical model framework. Findings also highlight that patients spend considerable time in the early stages following injury to understand and reflect on their injuries and accident. This study has also found that recovery does not always follow align with physical injury healing times. This is the first study capturing physiotherapists’ views specifically to musculoskeletal trauma, providing greater insight into challenges currently faced within healthcare and their potential influence on patient care.

This study is unique including all severity of injuries, whereas the majority of previous literature focuses to more major injuries only [40,46,47]. Findings suggest that injury severity and using timepoints aligned with physical injury healing times to assess and progress rehabilitation may not be helpful. Instead, focusing on individual needs at point of contact and referring to specialist services for all musculoskeletal trauma regardless of injury severity would provide individualised patient centred care. Current NICE guidelines acknowledge that rehabilitation needs may not correlate with injury severity should focus on patient needs at the time of assessment [4]. However, these guidelines focus on the initial rehabilitation phase during the inpatient stay rather than at any point of contact following injury, leaving the current model of follow up aligned with physical injury healing times [4]. While appropriate for progressing weight bearing status following a lower limb fracture for example, this model of care is more focused to a medical model than holistic patient centered approach. This medicalised model approach therefore has the potential to lose focus on the emotional response to injury and recognising when this response is prolonged and unhelpful to recovery [15,48]. The findings support the importance of supporting patients both physically and emotionally, with previous studies demonstrating that psychological support is often limited [48]. Additionally, patients described the feeling of losing their ‘safety net’ upon discharge and the significant amount of time participants spent during the interviews discussing and processing the injuries and accident. The need for support following discharge has been reported previously from patients with musculoskeletal trauma both physically or emotionally [13,49,50]. Access to healthcare support was often not a positive experience shifting reliance to family and friends with previous literature supporting this finding [51]. Evidence suggests in both musculoskeletal trauma and other conditions such as traumatic brain injury that intensive rehabilitation can be effective for long-term recovery [52–55]. However, most studies focus on the inpatient stay rather than upon discharge. Additionally, due to limited resources within the NHS, intensive rehabilitation may not always be possible in all areas of the country and nations. The results highlight care provision shortcomings and discharge is described as quick with limited support. There is a need to investigate cost effective ways of supporting patients, especially within the first few months following injury when support is needed rather than just at point of physical injury healing time reviews, i.e., fracture clinic. Examples of novel ways in healthcare of supporting patients upon discharge include digital health interventions such as remote monitoring or telehealth [56,57] and could be considered in the future for this cohort of patients.

The complexity of recovery was highlighted in the physiotherapist focus groups with no consensus on the most appropriate outcome measure or what could be used to show recovery progress. Currently, there is no recommended core outcome set for the musculoskeletal trauma population, despite some having been developed for specific injuries such as open lower limb fractures [58], highlighting the challenges in developing suitable outcome measures for this population.

### Coherence between patient and physiotherapist perspectives

This is the first study to explore and compare the views and perceptions of physiotherapists and patients specifically related to musculoskeletal trauma. Multiple themes illustrate consistencies across the patient interviews and physiotherapist focus groups. For example, factors influencing recovery such as psychological impact, fear of reinjury and the period of adjustment and acceptance were discussed in both focus groups and patient interviews as well as the system pressures affecting recovery. However, views on what successful recovery meant to patients, and what physiotherapists felt was important to patients differed. A significant difference highlighted is that communication was continually discussed within the focus groups as critical to recovery, whilst the patient interviews highlighted this was often not the case. Whether communication was between MDT members and the patient, or between MDT members it was highlighted as lack of consistency in messages to the patient. This could be due to multiple factors. Firstly, hospital pressures could impact the care patients received overall, and this could therefore potentially impact on communication to the patient. This has been reported in the literature in other conditions such as stroke [59]. Secondly, in the early stages of recovery patients are processing their injuries and experiencing potential side effects of medication and pain [6,60], which could affect how well the patient can understand and retain information in this early stage of recovery. Exploring how best to communicate key information to patients during the early stages of recovery and following discharge could allow better understanding and engagement in rehabilitation despite potential barriers such as high pain intensity.

Another noteworthy finding is that injury severity was seen as being a factor which can influence recovery and what patients could achieve, with physiotherapists feeling the injury severity should dictate rehabilitation following injury. This contrasts with patients’ views who wanted to return to ‘normal’ regardless of injury severity. This aligns with findings in a study focused to shoulder problems, where patient definitions of successful recovery varied and focused to meaningful activities, whilst physiotherapist definitions were focused on when the patient could ‘manage’ on their own [19]. Although direct comparisons cannot be made as this study was focused to shoulder problems, it highlights differing views between patient and physiotherapist around recovery and highlights a wider problem within physiotherapy practice. However, it is unclear why patient and physiotherapist views do differ. A potential explanation could be the influence of preconceived ideas or unconscious bias which have been formed through education and clinical experience [61,62]. This could explain why physiotherapists perceive they are addressing patient needs but patient experience differs. Unconscious bias can influence how healthcare interact with patients and influence decision making with detrimental effects [61–63]. Whilst existing research on unconscious bias often focuses on ethnicity, gender, age etc [61], little is known about it’s impact on injury perceptions and clinical decision making. Further research is needed around unconscious bias in clinical decision making, and how to support clinicians in managing patients following musculoskeletal trauma. Developing of a framework which could assist clinicians in how they can manage difficult conversations and potentially manage any known pre-conceived ideas such as injury severity could be useful.

### Strengths and limitations

A strength of this study is that it incorporates both patient and physiotherapist views, providing greater insight into the early stages of recovery from a service level as well as patient experience. This study also explores the breadth of injury severities rather than focusing on major injuries. Interviews were conducted at an early timepoint (5–44 days post injury) to capture participants’ initial thought processes on their recovery as it is occurred making this research novel. Further research is now required to compliment these findings at later timepoints in a longitudinal approach to understand the recovery journey in its entirety. Alongside the patient views, this study has captured physiotherapists views allowing a more comprehensive understanding of recovery and rehabilitation following musculoskeletal trauma.

Patient and public involvement has been integral to this study from inception with strategies to ensure trustworthiness, such as participants reviewing transcripts following interviews and focus groups. Although participants could have reviewed the synthesised findings to further enhance the trustworthiness of the data, this was a pragmatic decision a-priori following discussion with PPI groups to avoid overburdening participants during this difficult period of transition following the injury.

A limitation of this study was the physiotherapists were only from an inpatient setting. While some of these clinicians did have outpatient experience, this was not their current focus. Considerable efforts were made to recruit outpatient staff, but due to the timing of the study, staff capacity to participate was restricted. This was discussed within the Study Steering Group and Study Management Group, leading to the pragmatic decision to stop recruiting for the focus groups. Future studies incorporating outpatient physiotherapists’ views on recovery would be beneficial. Additionally, although focus groups are useful for generating discussion and gaining different viewpoints, there is the risk that dominant voices within the group could limit alternative views, and could be seen as a limitation of this method. However, every effort was made to reduce thus risk, including having a moderator for the focus group and setting out expectations at the beginning of the group that we are all views were of interest.

Another limitation is that while every effort was made to complete the interviews within four weeks of injury, this was sometimes not possible for all participants due to medical procedures, and the patient preference to complete the interview at home upon discharge for privacy. From the analysis completed, this did not impact the results and themes presented and provided additional insight into the few weeks following discharge, which might not have been captured otherwise. Future studies should consider capturing data within six weeks of injury to allow adequate participation and rich data. Additionally, it is recognised that this is a challenging time for participants, reflected in that two participants withdrawing from the first interview due to feeling unable to discuss their accident/injuries. While these additional insights are not reflected within this study, they highlight the importance of support and guidance for patients in the early stage of recovery.

Finally, all interviews and focus groups were conducted face to face online, with one interview being conducted via the telephone. Face to face interviews in person were not possible due to the COVID pandemic. However, video calling allowed observation of body language and collection rich, insightful data. Although one telephone interview was conducted without observing body language, this participant could not access video calling technology. It was decided a-priori to include telephone interviews as an option to be inclusive and representative of the population.

## Conclusions

The early stages of recovery are complex and individual to the patient. Recovery does not follow injury healing trajectories and is irrespective of injury severity, age or gender. Considerable time in the early stages following injury is processing the injuries and accident with a clear definition of successful recovery is returning to normal. Multiple influences impact on the early stages of recovery. Differences in key messages between the patients and physiotherapists have been highlighted. This highlights the need for effective communication and a development of a framework which would support all members of the multi-disciplinary team in supporting patients in their recovery. Future studies exploring patient views of their recovery at later stages of their journey are now required.

## Supporting information

S1 File Supplementary File 1: Interview and Focus Group Audit Trail. (DOCX)

S2 File Supplementary File 2: COREQ Checklist. (PDF)
