# Correction: Experiences, views and perceptions of recovery following musculoskeletal trauma of patients and physiotherapists: A qualitative study

**DOI:** 10.1371/journal.pone.0339048

**Published:** 2025-12-16

**Authors:** The PLOS One Staff

There are errors in the author affiliations. The correct affiliations are as follows

Nicola Middlebrook^1^, Nicola R. Heneghan^2^, Maria Moffatt^3^, Lucy Silvester^4^, Deborah Falla^6^, Alison B. Rushton^5^, Andrew A. Soundy^2^

**1** Department of Health Professions, Faculty of Health and Education, Manchester Metropolitan University, Manchester, United Kingdom, **2** School of Sport, Exercise and Rehabilitation Sciences, College of Life and Environmental Sciences, University of Birmingham, Birmingham, United Kingdom, **3** School of Allied Health Professions and Nursing, Institution of Population Health, University of Liverpool, Liverpool, United Kingdom, **4** Institute for Applied & Translational Technologies in Surgery, University Hospitals Coventry and Warwickshire NHS Trust, Clifford Bridge Road, Coventry, United Kingdom, **5** Western University, School of Physical Therapy, London, Ontario, Canada, **6** Centre of Precision Rehabilitation for Spinal Pain, School of Sport, Exercise and Rehabilitation Sciences, College of Life and Environmental Sciences, University of Birmingham, Birmingham, United Kingdom

The publisher apologizes for the error.
