# Supplementary material for: Experiences, views and perceptions of recovery following musculoskeletal trauma of patients and physiotherapists: a qualitative study
Source: PLoS One. 2025 May 28;20(5):e0323575. doi: 10.1371/journal.pone.0323575 (PMC12119110; doi:10.1371/journal.pone.0323575)
Supplement: S1 File — (DOCX) [file pone.0323575.s001.docx]

S1: Patient Interview and Focus Group Audit Trail

| Stage | Details of process | Example action point |
| --- | --- | --- |
| Pre-Analysis | SMG progress meetings | Discussed current data collection. Recruited targeted 20 participants but some not completed interview. SSG reviewed patient characteristics and initial views around data (richness and thickness of data). Deemed to recruit further 5 participants to meet sampling criteria e.g. more female.  Reviewed again after additional recruitment, sample themes from initial reading of transcripts discussed. SMG agreed sufficient data to proceed with further more in depth analysis no further recruitment needed. |
| Data analysis – IPA Stage 1 - Reading transcripts and note making | NM and MM read all transcripts independently and notes documented on initial thoughts  Initial thoughts discussed particularly any thoughts which contrasting or could add further detail to themes/subthemes | “No, I think it's all the same. I think I just have to keep a positive outlook of what I can do to make it better. I mean, I keep telling myself in one sense I'm going to be 100% again. But in the back of my mind, it's like well, if I put 200%, a bit more effort in, the chance that I could get it stronger than before. I could build upon it, build the muscle within my leg stronger than ever before, then that might be more beneficial to me than it was before” **NM Comment:** Recovery definition - physical and mental. **MM Comment**: And that recovery doesn't always mean being 100% as you were pre-injury.  ACTION: both comments added to mindmap for preliminary themes |
| Data Analysis - IPA Stage 2 Preliminary themes and coded as per IPA | Preliminary themes coded by NM and reviewed by MM  Critical friend (AS) reviewed initial themes and condensing of themes to 3 main themes with subthemes. | Initially there were approximately 6-7 main themes. Using a mindmap and discussion of themes with AS the themes were rearranged into 3 main themes and subsequent subthemes and combined where possible e.g. emotion initially one main theme but these were combined and intertwined with themes : understanding accident/injuries and the early stages of recovery. |
| Data Analysis – IPA Stage 3 – Themes and codes presented to SMG | All themes and subthemes with example quotes presented to SMG. Any proposed changes were made by NM and presented to SMG until all were happy with themes/codes. | Subtheme 4 – experience of healthcare following trauma  Change subtheme name to reflect:  Patient no where near the centre of care  Managing injuries rather than the patient.  Managing fractures rather than the patient  ACTION: Subtheme changed to Shortfall of patient centred care and management of injuries rather than the individual. |
| Data Analysis – IPA Stage 4 – Themes and codes presented to SSG | All themes and subthemes with example quotes presented to SSG. Any proposed changes made by NM and circulated to SMG following meeting. | Recovery guided by healthcare professionals  is this more to do with control?  Is it guided by or more being told?  Look at quotes and see if need to adjust subtheme  ACTION – Combination of being guided than being told, no change in subtheme name. |

Focus Group Audit Trail

| Stage | Detail of process | Example action point |
| --- | --- | --- |
| Pre-Analysis | SMG progress meeting | Discussed lack of outpatient physiotherapists and strategies employed to recruit. Acknowledged issues with hospital pressures main problem. Discussed grant timelines and current data. Agreed to progress with data analysis. |
| Stage 1 – Reading of transcripts and note making | NM and NH read transcripts and made initial notes to discuss. | N/A |
| Stage 2 – Initial framework developed | NM developed initial framework and discussed with NH. | Discussion around initial themes. Initial themes were very broad. E.g. V1 ‘Recovery Concept’ changed to ‘Process of Recovery’ |
| Stage 3 – Development of framework with example quotes and presented to SMG | NM further developed framework with NH and presented to SMG. Any proposed changes were made by NM and presented to SMG until all happy with themes/codes | Every trauma survivor is impacted psychologically  Is this correct term trauma survivor? Survivor of trauma?  ACTION: Changed to Every survivor of trauma is impacted psychologically. |
| Stage 4 – Themes and codes present to SSG | All themes and subthemes with example quotes presented to SSG. Any proposed changes made by NM and circulated to SMG following meeting. | Subtheme what is the endpoint of recovery  Term endpoint not liked. Change back to successful recovery? What is recovery? Or What is successful recovery? What is being recovered?   Does recovery have an endpoint? Endpoint of time moreso.   Commissioner perspective – they will see the endpoint of time.   ACTION: Subtheme adjusted to ‘what is being fully recovered’ |
